# Outcomes in Hospitalized Patients With Subclinical Hypothyroidism

**DOI:** 10.7759/cureus.91051

**Published:** 2025-08-26

**Authors:** Mayesha Sharaf, Pinak Shah, Andrey Manov, Kartika Shetty, Rakahn Haddadin

**Affiliations:** 1 Internal Medicine, MountainView Hospital, Las Vegas, USA

**Keywords:** euthyroid, in-hospital mortality, length of stay, major adverse cardiovascular events, readmission, subclinical hypothyroidism

## Abstract

Background

This study aimed to investigate the relationship between subclinical hypothyroidism (SCH) and the occurrence of major adverse cardiovascular events (MACE) amongst hospitalized patients. The study further explored the association between SCH and the patients’ in-hospital mortality, average length of stay (LOS), and 30-day readmission for all causes.

Methods

This was a retrospective, multi-center study. Patients with both thyroid-stimulating hormone (TSH) and free thyroxine (T4) levels available were included and categorized as euthyroid if TSH was 0.36-3.74 mIU/L with normal free T4 and subclinical hypothyroid if TSH was 3.75-9.99 mIU/L with normal free T4. Patients with abnormal free T4 levels, a history of hypothyroidism or hyperthyroidism, and use of any thyroid medications were excluded. Outcomes studied were MACE, in-hospital mortality, hospital LOS, and 30-day readmission for all causes.

Results

Among the group, 13.4% of the euthyroid group had a MACE event, compared to 14.2% in the SCH group. Mortality rate, 30-day readmission, and average LOS were 3.8%, 20.3%, and 4.80 days in the euthyroid group and 5.9%, 23.2%, and 4.89 days in the SCH group, respectively. Logistic regression models for the outcome MACE did not show statistical significance between the euthyroid and SCH groups. No statistically significant associations were found in secondary outcomes either.

Conclusion

Our study showed increased MACE, higher mortality, higher 30-day readmission, and prolonged LOS in patients with SCH compared to euthyroid patients. However, the difference was not statistically significant. Our study supports the importance of further prospective studies to better understand the clinical implications of SCH in hospitalized patients and to refine management and treatment strategies.

## Introduction

Hypothyroidism is a clinical condition caused by an underactive thyroid gland. It is diagnosed when low levels of thyroid hormones result in elevated levels of thyroid-stimulating hormone (TSH). Subclinical hypothyroidism (SCH), also called mild thyroid failure, is a condition where thyroid hormone levels are within the normal reference laboratory range, but TSH is mildly elevated. It occurs in 6.2% of women over the age of 60 and 3.6% of elderly men [1, 2]. Although this condition can be asymptomatic to mildly symptomatic, it correlates with fatal and non-fatal major adverse cardiovascular events (MACE) [3]. SCH is common in the hospital setting as well, whether it is secondary to the patient’s chronic condition or a primary condition that was undiagnosed.

Numerous studies have shown the association of SCH with adverse cardiovascular events. It could be due to the detrimental effects of SCH on blood pressure or lipid levels. SCH also increases the risks of metabolic syndrome and atherosclerosis, further associating it with cardiovascular diseases. As cardiovascular diseases already account for significant mortality and morbidity worldwide, it is imperative to identify factors that can accelerate such processes to reduce adverse cardiovascular events. Thyroid hormone has been shown to modulate cardiac function, including contractility and vascular resistance [4]. Thyroid hormone regulates the synthesis of proteins that contribute to the lusitropic, dromotropic, and chronotropic effects of the heart by influencing calcium influx into myocytes [5]. Diastolic dysfunction has been shown to be the most frequent cardiac impairment in patients with SCH [6]. 

The coexistence of SCH with hypertension (HTN) is beyond doubt. Hypothyroidism is associated with increased systemic vascular resistance (SVR) and serum low-density lipoprotein (LDL) level [7, 8]. A study conducted in China showed a positive association between SCH and the prevalence of HTN. The study also showed that the association was even more profound in females and individuals aged < 65 years [9]. The correlation between SCH and dyslipidemia is also well established. There are numerous studies that showed a positive association between SCH and dyslipidemia [10-12]. A study conducted in Saudi Arabia in 2023 showed a correlation between SCH and metabolic syndrome [13]. The association between SCH and abnormal cholesterol or triglyceride levels is, however, not well established [14]. SCH also alters thermogenesis and metabolic rate and thus is associated with an increase in visceral adipose tissue, especially thoracic periaortic adipose tissue, body roundness index, and Chinese visceral adiposity index [15]. 

SCH is also associated with insulin resistance and impaired coagulation by increasing thrombotic risks, which can further increase the risk [16, 17]. Low normal thyroid function is shown to be associated with insulin resistance and, thus, increased cardiovascular risk [18]. SCH also decreases the insulin sensitivity index [19]. Moreover, a study conducted in China established that SCH is related to a prothrombotic state, which could be due to alterations in coagulation and fibrinolysis [20]. Based on its findings, this study recommended screening for SCH for detecting the earliest signs of cardiovascular diseases.

Another study conducted in India showed impaired flow-mediated dilation in both SCH and overt hypothyroidism patients compared to euthyroid controls [21]. Impaired flow-mediated dilation is a preceding abnormality in the process of developing atherosclerosis [21]. SCH increases the risks of cardiovascular diseases via these abovementioned metabolic derangements. And SCH, being seemingly asymptomatic, can pose risks of MACE to the patients while remaining completely unknown to them.

Whether to treat SCH or not remains controversial. As hypothyroid symptoms are unspecific by nature, the decision to treat or not has to be individualized [22]. Factors such as age, ethnicity, body mass index, and smoking should be considered. Most guidelines recommend treatment if TSH > 10 mIU/L [23]. However, more studies are necessary to evaluate the importance of initiating treatment of SCH with levothyroxine in hospitalized patients, even in the setting of TSH < 10 mIU/L, especially in patients with known cardiovascular risk factors, to prevent MACE and to reduce long- and short-term mortality.

## Materials and methods

Subjective

The aim of our study was to investigate the relationship between SCH and the occurrence of MACE in hospitalized patients. The study will further explore the associations between SCH and the patients’ in-hospital mortality, average length of stay (LOS), and 30-day readmission. 

Data source

Our study is a retrospective, multi-center study that was conducted using data from the Hospital Corporation of America (HCA) Healthcare nationwide database between January 1, 2017, and December 31, 2019. The protocol was submitted to the Institutional Review Board (IRB) of MountainView Hospital, Las Vegas, NV, and the research activity was determined to be exempt from IRB oversight in accordance with institutional regulations and policies, as it was a retrospective study. Laboratory values and events of interest in qualified participants were collected through retrospective chart review while preserving privacy by means of de-identified data collection.

Study design

Patients above 18 years old and who had both TSH and free thyroxine (T4) levels available during admission in enterprise-wide hospitals throughout the country from January 1, 2017, to December 31, 2019, were identified and included. The first available TSH and free T4 levels during that hospitalization were used for the analysis. From the initial encounter, we excluded patients who had a free T4 level < 0.76 ng/dL or > 1.46 ng/dL and TSH < 0.36 mIU/L or > 10 mIU/L. Patients with any pre-existing history of hypothyroidism, hyperthyroidism, or use of any thyroid medications (levothyroxine, Synthroid, Armour Thyroid, methimazole, or propylthiouracil) were excluded from the study as well. Patients who met the inclusion and exclusion criteria were then categorized into two groups: the euthyroid group if TSH was 0.36-3.74 mIU/L and the SCH group if TSH was 3.75-9.99 mIU/L (Table 1).

**Table 1:** Patient categories based on thyroid-stimulating hormone (TSH) level

Categories	TSH level
Euthyroid group	0.36-3.74 mIU/L
Subclinical hypothyroidism group	3.75-9.99 mIU/L

Baseline characteristics were obtained, including age, sex, race, tobacco use, BMI, HTN, diabetes mellitus (DM), chronic obstructive pulmonary disease (COPD), chronic kidney disease (CKD), end-stage renal disease (ESRD), and congestive heart failure (CHF). The primary outcome was MACE (myocardial infarction, unstable angina, ischemic stroke, transient ischemic attack, and critical limb ischemia). To have a complication recorded, diagnostic codes must not be present on admission (Appendix A). Secondary outcomes were in-hospital mortality, average LOS, and 30-day readmission. 

Statistical analysis

A logistic regression model was used to obtain an odds ratio (OR) to predict the likelihood of binary variables, which included MACE, in-hospital mortality, and 30-day readmission. ORs for the above-mentioned outcomes were calculated while keeping the baseline characteristic constant to minimize confounding biases. A negative binomial regression model was used to ascertain the incidence rate ratio (IRR) for LOS, which is similar to the OR for the logistic regression model. The baseline characteristic variables used in the model were age, sex, race, tobacco use, BMI, HTN, DM, COPD, CKD, ESRD, and CHF. The use of logistic regression allowed us to analyze multiple explanatory variables and reduce confounding effects. 

## Results

The retrospective chart review initially gathered 27,389 encounters. Following initial stratification and application of exclusion criteria, 11,118 patients remained in the final data set. 52.1% of the patients were female, and 47.9% of the patients were male; 86.7% of the patients were categorized in the euthyroid group, and 13.3% were categorized in the SCH group. Table 2 presents detailed demographic information.

**Table 2:** Patient demographics *‘Others’ includes racial and ethnic groups such as Hispanic, American Indian, Native Hawaiian, Asian, Southeast Asian, and Middle Eastern.

Variables	Number of patients (N)	N%
Female	5,791	52.1%
Male	5,327	47.9%
Race: Black	1,576	14.2%
Race: White	6,135	55.2%
Race: Others*	3,407	30.6%
Euthyroid Group	9,637	86.7%
SCH group	1,481	13.3%
Hypertension	6,612	59.5%
Diabetes mellitus	3,241	29.2%
Chronic obstructive pulmonary disease	1,361	12.2%
Chronic kidney disease	1,395	12.5%
End-stage renal disease	398	3.6%
Congestive heart failure	2	0.0%

MACE

MACE occurred in 1,294 patients out of 9,637 patients in the euthyroid group (13.4%) and 211 patients out of 1,481 patients in the SCH group (14.2%). While MACE occurred more frequently in the SCH group, the logistic regression model for the outcome did not show a statistically significant difference between the euthyroid and SCH groups when the control variables were held constant (OR=1.03, 95% CI (0.87 - 1.21), p=0.722).

In-hospital mortality

Out of 9,637 patients in the euthyroid group, 369 patients experienced in-hospital mortality (3.8%) compared to 87 patients out of 1,481 patients (5.9%) in the SCH group when the control variables were held constant (OR=0.77, 95% CI (0.60-1.00), p=0.050). In-hospital mortality was more common in the SCH group; however, logistic regression analysis showed no statistically significant difference.

Average LOS

The average LOS was 4.80 in the euthyroid group and 4.89 in the SCH group. Negative binomial regression analysis showed no statistically significant difference between the two groups when the control variables were held constant (OR = 1.04, 95% CI (0.97-1.10), p = 0.215).

Thirty-day readmission

Thirty-day readmission rates were higher in the SCH group. Out of 1,481 patients in the SCH group, 344 were readmitted within 30 days (23.2%); 1,959 patients out of 9,637 patients in the euthyroid group were readmitted within 30 days (20.3%). However, the difference was not statistically significant (OR=0.87, 95% CI (0.76-1.00), p=0.051) per logistic regression analysis with the control variable held constant.

Table 3 demonstrates the events in between the euthyroid and SCH groups. Table 4 demonstrates the predicted likelihood of variables between the euthyroid and SCH groups. 

**Table 3:** Frequency of outcomes between two groups N%: percentage of the group related to the corresponding event

Events	Euthyroid group (N%)	Subclinical hypothyroidism group (N%)
Major adverse cardiovascular events	13.4%	14.2%
In-hospital mortality	3.8%	5.9%
Average length of stay (days)	4.80	4.89
30-day readmission	20.3%	23.2%

**Table 4:** Predicted likelihood of variables in euthyroid and subclinical hypothyroidism group by logistic regression P-value is considered significant if <0.05. Logistic regression was used to calculate the p-value of MACE, in-hospital mortality, and 30-day readmission. Negative binomial regression was used to calculate the p-value of the average length of stay.

Outcomes	Odds ratio	95% confidence interval	P-value
Major adverse cardiovascular events (MACE)	1.03	0.87-1.21	0.722
In-hospital mortality	0.77	0.60-1.00	0.050
Average length of stay	1.04	0.97-1.10	0.215
30-day readmission	0.87	0.76-1.01	0.051

## Discussion

Our study has shown a higher incidence of MACE in patients with SCH compared to euthyroid patients. However, the correlation was not statistically significant. This study also indicated higher in-hospital mortality, 30-day readmission rates, and longer LOS in the SCH group. However, like the primary outcome, these correlations were also not statistically significant. Nonetheless, our findings are consistent with the recent studies on the correlation between SCH and MACE, suggesting increased MACE in the SCH group.

The correlation between SCH and MACE has been studied by numerous meta-analyses. The prevalence of adverse cardiovascular events in patients with and without SCH was investigated by a study conducted in Australia. Higher statistical significance in both cross-sectional and longitudinal analyses (age- and sex-adjusted hazard ratio (HR) 1.7; 95% CI 1.2-2.5; p<0.01) was reported in SCH patients compared to their euthyroid controls [24]. The risks of adverse cardiovascular events remained significantly higher even after adjusting for the standard cardiovascular risk factors. Another meta-analysis illustrated that SCH is associated with significant adverse cardiovascular events at baseline and at follow-up (relative risk (RR) with 95% CI 1.533 (1.312-1.791); p<0.05 and RR with 95% CI 1.188 (1.024-1.379); p<0.05, respectively) [25]. The differences in 95% CI observed across these studies have been associated with variations in study designs, the severity of SCH entailed by the value of TSH and the severity of symptoms of SCH, and the gender and age of the participants. 

A longitudinal study conducted in Taiwan followed 11,574 patients for 10 years. The participants did not have any known history of thyroid disorder; however, 1.6% of the participants had SCH. The study showed that 680 out of 3,669 deaths were due to MACE [26]. The relative ratio of deaths from all causes and MACE was higher in the SCH group compared to the euthyroid group, even after the confounders were adjusted. This study concluded that Taiwanese patients with SCH have an increased risk for all-cause mortality and MACE [16] [26].

A study done in Tel Aviv in 2020 to demonstrate the association between unknown SCH and in-hospital outcomes among patients with ST-segment elevated myocardial infarction had shown that undiagnosed SCH was independently associated with increased likelihood of developing acute kidney injury and lower left ventricular ejection fraction, leading to poor in-hospital outcomes. SCH was also associated with higher short-term and long-term mortality following ST-segment-elevated myocardial infarction compared to euthyroid patients [27]. These findings comply with numerous other studies that have established that patients with cardiovascular disease and concomitant SCH showed poorer outcomes, including higher mortality and complications, compared to those without SCH. A study conducted in Japan demonstrated that patients with heart failure and coexisting SCH had lower peak breath-by-breath oxygen consumption, higher mean pulmonary arterial pressure, and increased risk of all-cause mortality during three-year follow-up [28]. Another longitudinal study with 12 years of follow-up reported increased cardiovascular events and mortality among adults with SCH and concomitant cardiovascular risk; patients younger than 65 years of age were at higher risk [29]. A meta-analysis of 35 studies showed all-cause mortality was greater in patients with SCH. The risk was even higher in the subgroup with high cardiovascular risk after correction for the confounding factors [30].

The strength of our study lies in the large sample size; 11,118 patients were included in the study, where data were collected from the nationwide HCA Healthcare hospitals’ database across the United States, which yielded a substantial sample size. Furthermore, to the authors' best knowledge, in-hospital outcomes of SCH-related MACE have not been assessed until now. The study conducted in Tel Aviv [11] evaluated patients with ST-segment-elevated myocardial infarction only. And a Taiwanese study [9] was conducted in screening centers. Our study is the first ever insight into how SCH affects mortality in the hospital and adds to the evidence of SCH associated with MACE. Further prospective studies are needed to analyze the effects of SCH on adverse cardiovascular events in hospitalized patients and the impact of treating SCH with a TSH level lower than 10 mIU/L. 

Our study has limitations as well. Although our study showed a positive association between SCH and MACE in the hospital, it was not statistically significant. Given the retrospective nature of the study, relevant information regarding possible history of SCH or use of thyroid medications could have been missing due to a lack of prior documentation. Also, TSH and free T4 levels are usually not routinely checked in hospital settings, rendering possible undiagnosed SCH in patients who had MACE in the hospital. Among the secondary outcomes, LOS and the 30-day readmission rate may often be related to the burden of chronic diseases and socio-economic conditions. In-hospital mortality could also be associated with the severity of chronic illness.

## Conclusions

This study has shown increased MACE mortality and 30-day readmission rates with prolonged LOS in hospitalized patients with SCH compared to euthyroid patients. However, the association was not statistically significant. Further studies are necessary to evaluate the impact of SCH on in-hospital MACE.

## Appendices

Appendix A

**Table 5:** Diagnosis codes Source: [31]

Diagnosis	International Classification of Diseases (ICD) code
Myocardial infarction	I21, I22, I23, I24
Unstable angina	I20.0
Ischemic stroke	I63
Transient ischemic attack	G65
Critical limb ischemia (native arteries)	I70.22, I70.23, I70.24, I70.26
Hypertension	I10, I11, I12, I13, I14, I15, I16
Diabetes mellitus	E08, E09, E10, E11, E13
Chronic obstructive pulmonary disease (COPD)	J41, J42, J43, J44
Chronic kidney disease (CKD)	N18
End-stage renal disease (ESRD)	N18.6
Congestive heart failure (CHF)	I150
